# Supplementary figures and images for: Maternal and zygotic gene regulatory effects of endogenous RNAi pathways
Source: PLoS Genet. 2019 Feb 13;15(2):e1007784. doi: 10.1371/journal.pgen.1007784 (PMC6391025; doi:10.1371/journal.pgen.1007784)

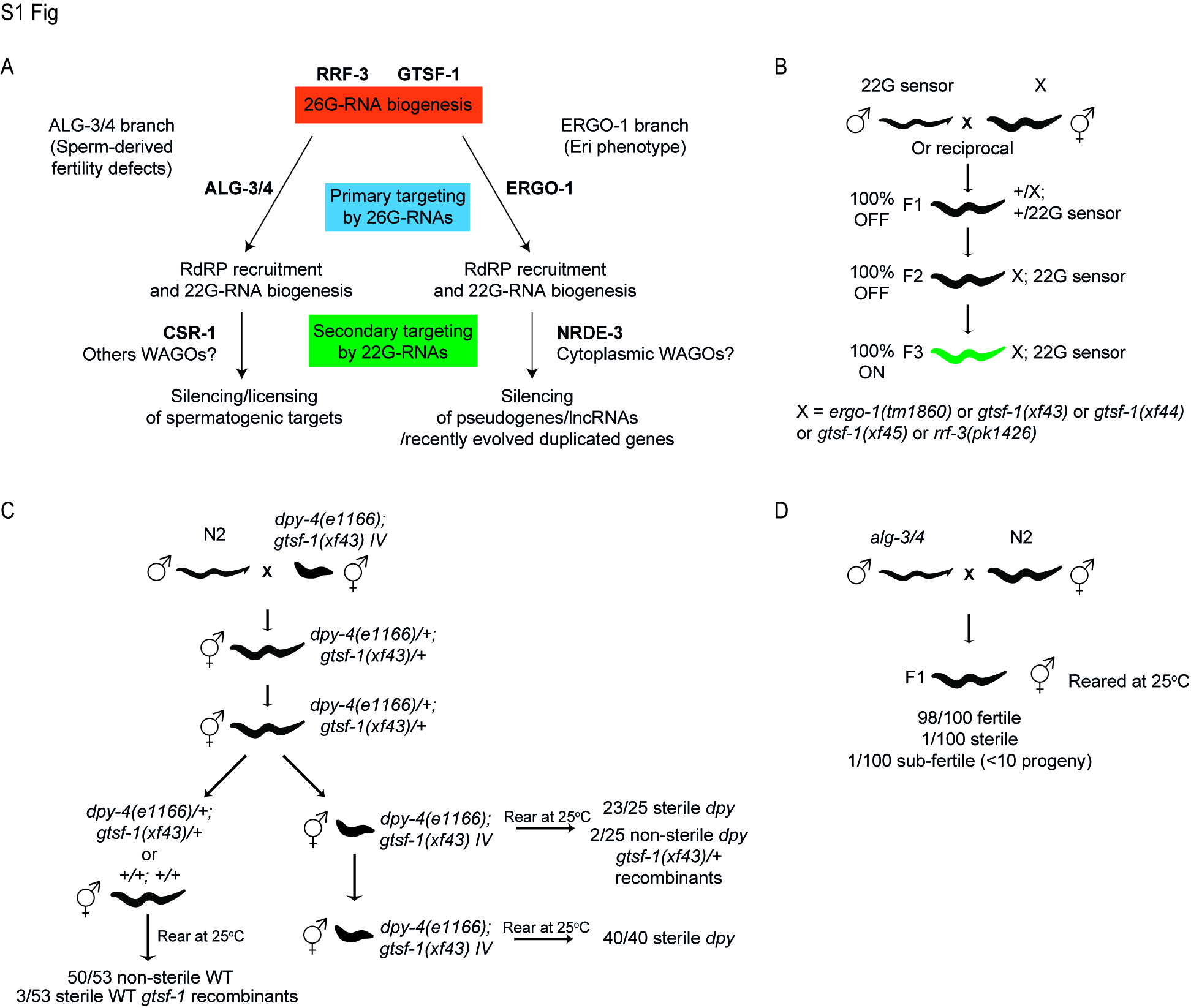

Supplement: S1 Fig — (A) Illustration of the current understanding of 26G-RNA pathways. 26G-RNAs are produced by RRF-3, assisted by GTSF-1 and other accessory factors. 26G-RNAs can associate with ALG-3/4 in the spermatogenic gonad (ALG-3/4 branch) or with ERGO-1 in oocytes and embryos (ERGO-1 branch). Upon target binding, RdRPs are recruited and synthesize secondary 22G-RNAs. NRDE-3 binds ERGO-1 branch 22G-RNAs, while CSR-1 is downstream of ALG-3/4 branch 26G-RNAs. Other unidentified Argonautes may play a role in these pathways. (B) Schematics of genetic crosses of mutant strains with the 22G sensor. Green worms illustrate derepression of the 22G sensor. Black worms depict repression of the 22G sensor. “X” corresponds to different mutant alleles that share the same maternal rescue. (C) Experimental setup to address the maternal transmission of the temperature-sensitive sterility phenotype at 25°C. Worms were constantly grown at 20°C until transfer to 25°C to assay sterility. L2-L3 worms were transferred to 25°C. (D) Experimental setup to test paternal effect by ALG-3/4 branch 26G-RNAs. alg-3/4 mutant males were crossed with wild-type hermaphrodites at 20°C. Cross progeny were isolated to fresh plates as L2-L3, transferred to and grown at 25°C. Fertility was assessed on the 3rd day of adulthood. (TIF) [file pgen.1007784.s001.tif]

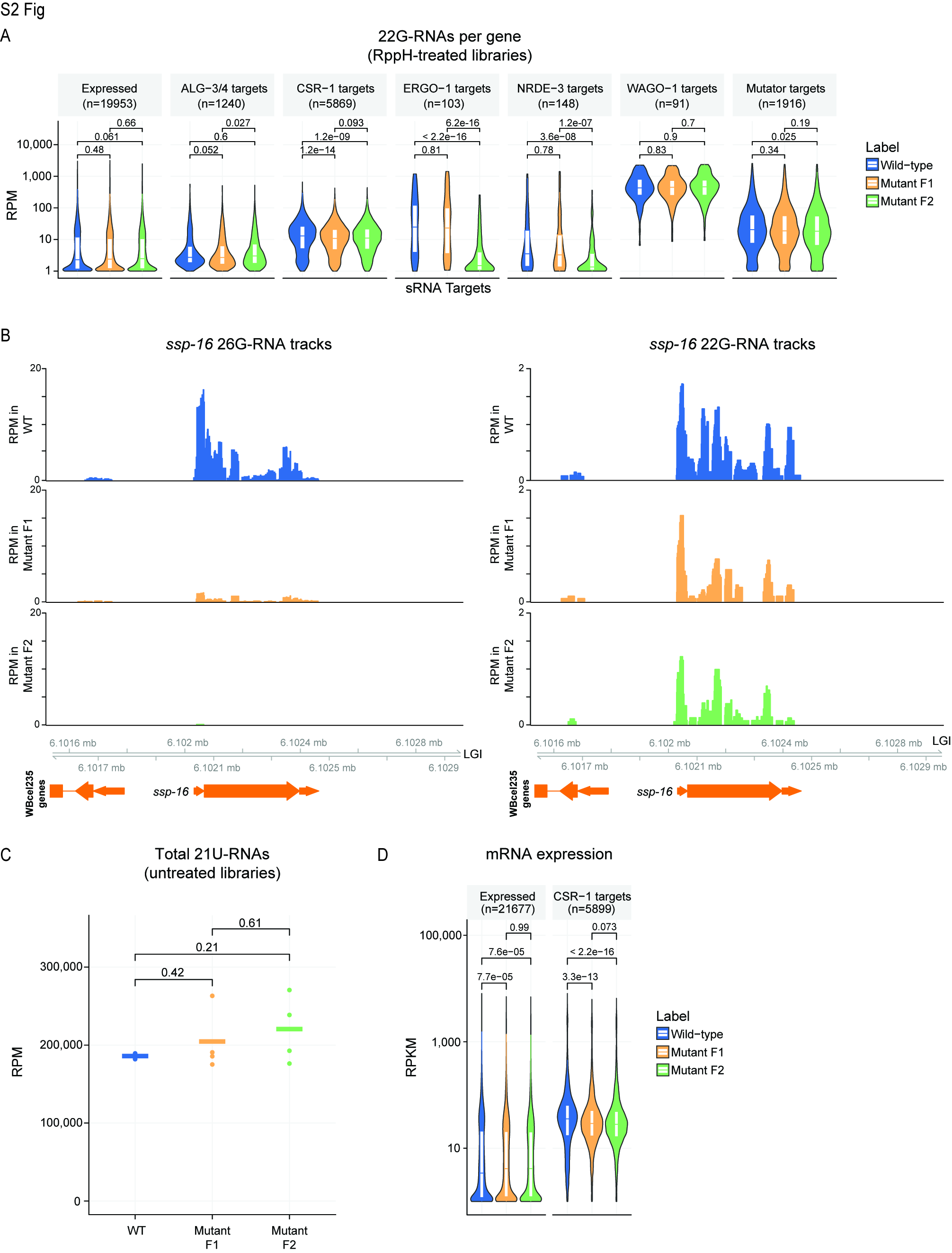

Supplement: S2 Fig — (A) Violin plot showing the distribution of RPM levels of 22G-RNAs mapping, per gene, to known targets of diverse sRNA pathways. Left-most panel shows the distribution of RPM levels in all genes with mapped 22G-RNAs. RPM values calculated from the RppH-treated libraries. (B) Genome browser tracks of ssp-16, a known ALG-3/4 target, showing mapped 26G- (left panels) and 22G-RNAs (right panels). 26G- and 22G-RNA tracks were obtained from untreated and RppH-treated libraries, respectively. (C) Total 21U-RNA levels in different generations/phenotype, in RPM. (D) Distribution of normalized mRNA expression of all expressed genes and CSR-1 targets, in RPKM. Violin plots in (A) and (D) show the distribution density of the underlying data. The top and bottom of the embedded box represent the 75th and the 25th percentile of the distribution, respectively. The line in the box represents the median. P-values were calculated with a two-sided unpaired Mann-Whitney/Wilcoxon rank-sum test. (TIF) [file pgen.1007784.s002.tif]

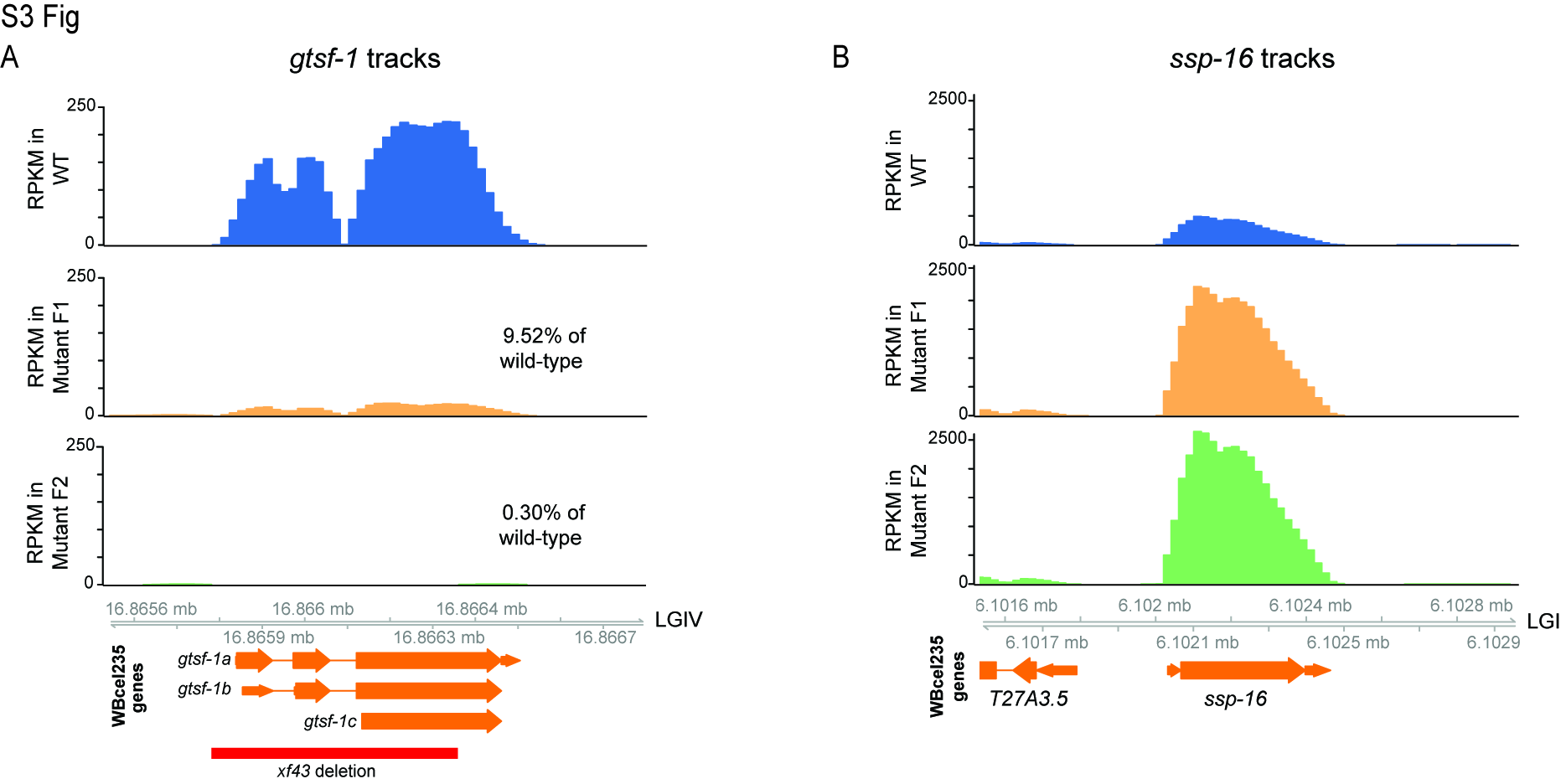

Supplement: S3 Fig — (A) Genome browser tracks displaying gtsf-1 mRNA levels in RPKM. The gtsf-1(xf43) deletion allele is represented below. gtsf-1 levels in the mutant F1 cover the xf43 deletion sequence, thereby indicating contamination with Dpy worms that recombined a wild-type copy of gtsf-1. The mutant F2 was isolated from mutant F1 Dpy whose gtsf-1 genotype was confirmed. Therefore, as expected, the only observed reads are flanking the xf43 deletion. (B) Genome browser tracks with the mRNA levels, in RPKM, of ssp-16. Upregulation occurs immediately in the F1, indicating no maternal effect. (TIF) [file pgen.1007784.s003.tif]

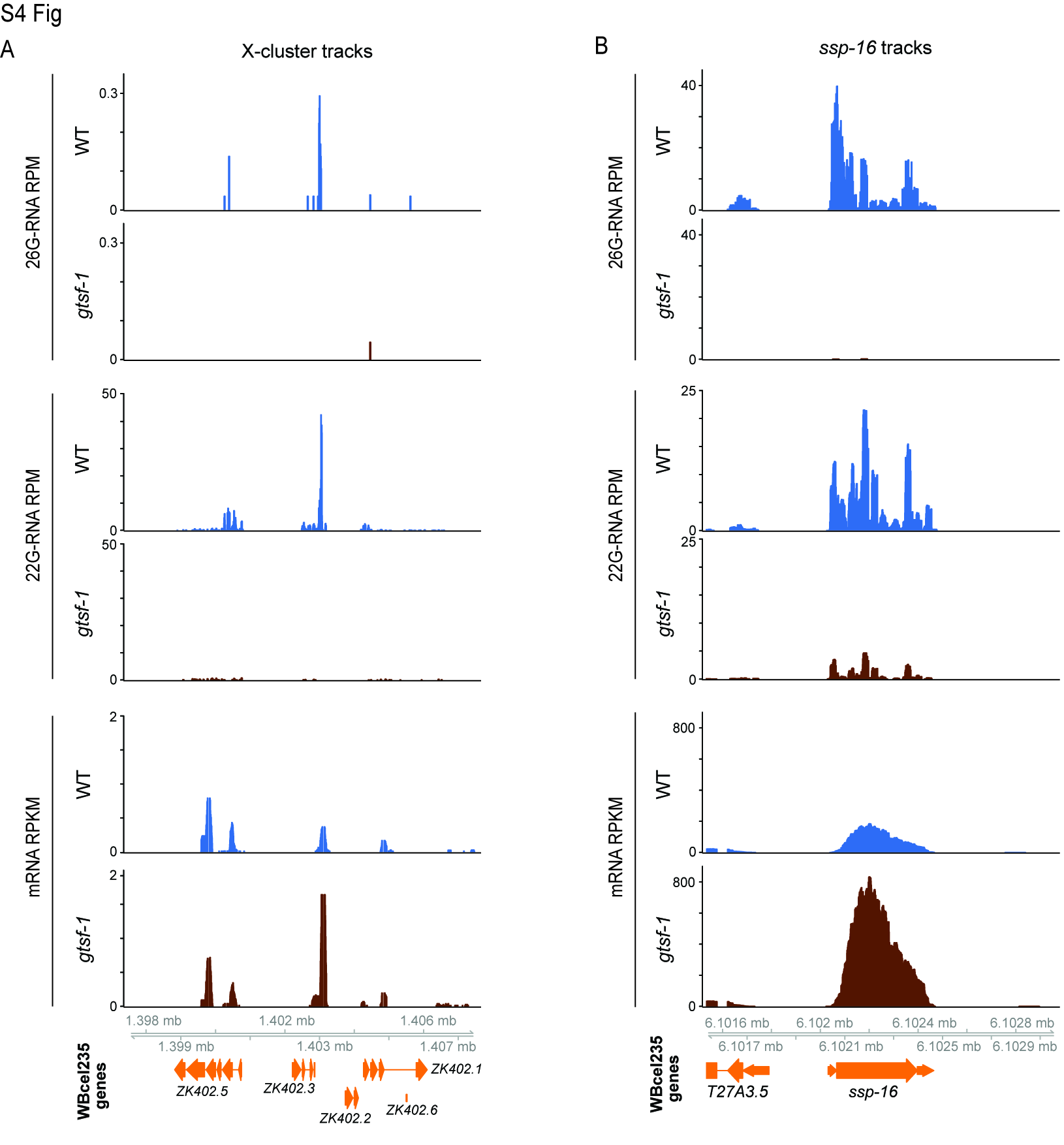

Supplement: S4 Fig — (A-B) RPM levels of 26G-RNAs (upper panels) and 22G-RNAs (middle panels) mapping to the X-cluster (A) and ssp-16 (B). Lower panels show RPKM mRNA levels of these targets. WT, wild-type. (TIF) [file pgen.1007784.s004.tif]

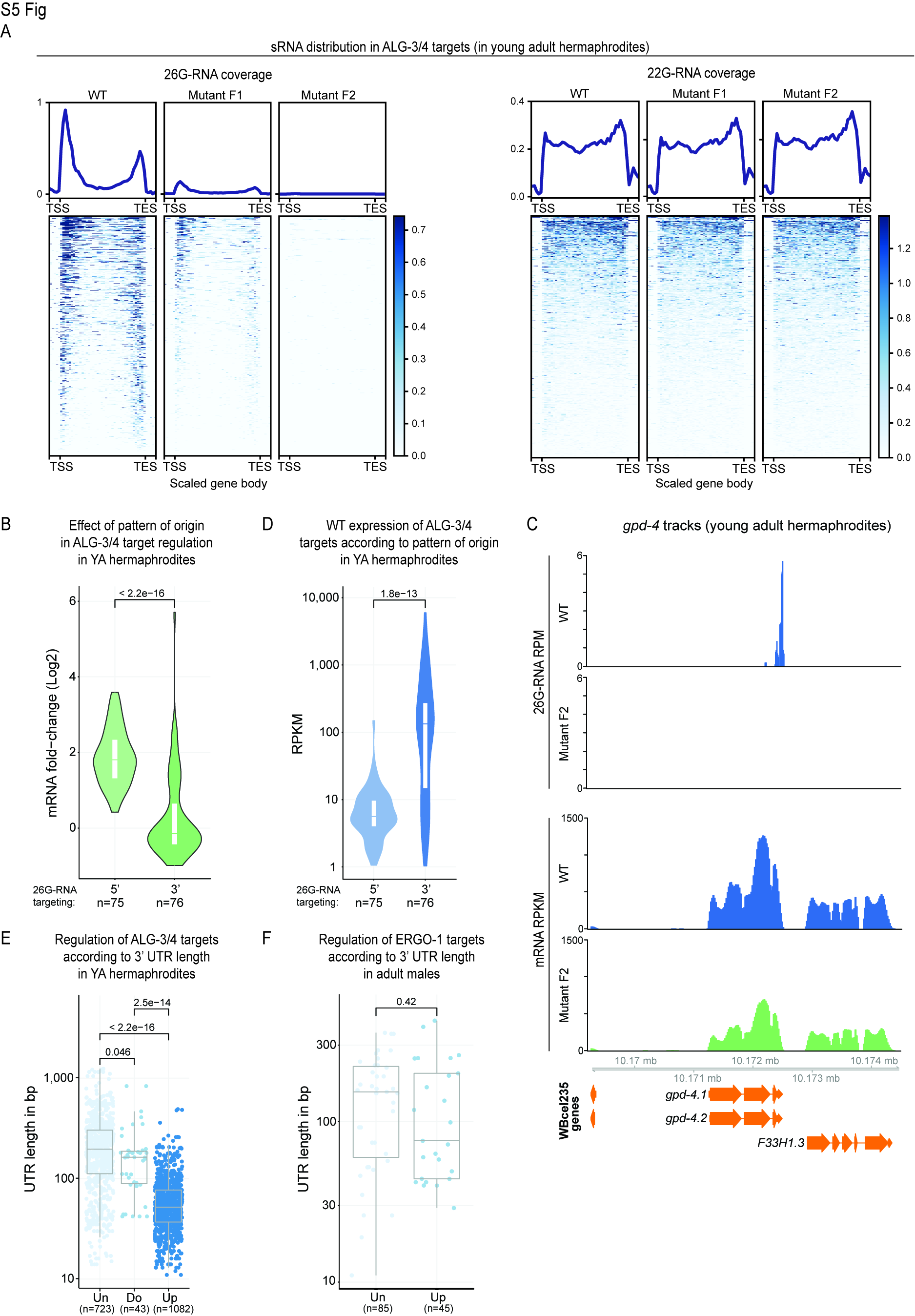

Supplement: S5 Fig — (A) Metagene analysis of 26G- (left panel) and 22G-RNAs (right panel) mapping to ALG-3/4 targets in young adult datasets, from our maternal effect setup (as in Fig 2A). On the upper part of each panel is the mean coverage profile for sRNA species in every generation. On the lower part of each panel, the heatmaps show the density across individual targets. Target gene body length was scaled between transcription start site (TSS) and transcription end site (TES). Moreover, the regions comprising 250 nucleotides immediately upstream of the TSS and downstream of the TES are also included. Simultaneous 26G-RNA targeting in the 5’ and 3’ can be observed in some genes. (B) Violin plot depicting the regulation of ALG-3/4 target genes predominantly targeted at the 5’ or at the 3’ by 26G-RNAs. (C) Genome browser tracks displaying the RPM levels of 26G-RNAs (upper panels) and RPKM mRNA levels (lower panels) mapping to gpd-4, a gene predominantly targeted by 26G-RNAs at its 3’ end, in young adult hermaphrodites. WT, wild-type. (D) Violin plot showing the wild-type expression levels of ALG-3/4 target genes predominantly targeted at the 5’ or at the 3’ by 26G-RNAs. (E) 3’ UTR lengths of all the transcript isoforms annotated for ALG-3/4 target genes, according to effect on gene expression. (F) 3’ UTR lengths of all the transcript isoforms annotated for ERGO-1 target genes, according to effect on gene expression. Fold-change data was obtained from adult male sequencing datasets. With the exception of (F), all the panels of this Fig were prepared using young adult hermaphrodite sequencing datasets from our maternal effect experiments. In B and E, regulatory outcome was defined as differential gene expression between the wild-type and gtsf-1 mutant F2. In (E-F), Un refers to genes with unchanged gene expression; Do means genes downregulated in the mutant; and Up refers to genes upregulated in the mutant. Violin plots in (B and D) and the boxplots in (E-F) show the distribution of the [file pgen.1007784.s005.tif]

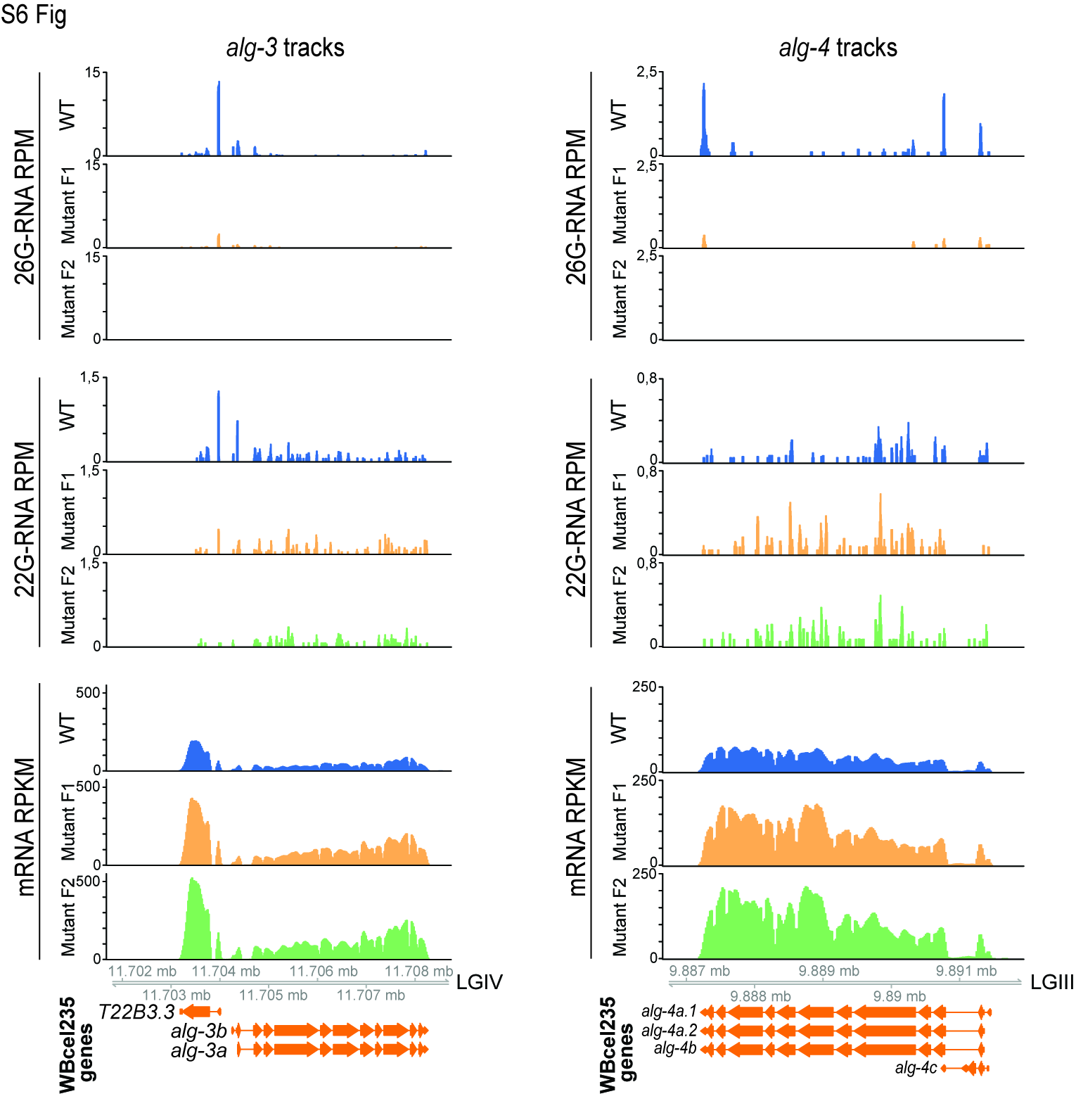

Supplement: S6 Fig — Genome browser tracks showing 26G-RNAs (upper panels) and 22G-RNAs (middle panels) mapping to alg-3 (left panels) and alg-4 (right panels), in RPM. Lower panels show the RPKM mRNA levels of alg-3 (on the left) and alg-4 (on the right). Sequencing datasets of young adult hermaphrodites from our maternal effect setup were used. (TIF) [file pgen.1007784.s006.tif]
